# Identification of Secreted O-Mannosylated Proteins From BCG and Characterization of Immunodominant Antigens BCG_0470 and BCG_0980

**DOI:** 10.3389/fmicb.2020.00407

**Published:** 2020-03-13

**Authors:** Guoying Deng, Wenli Zhang, Na Ji, Yunpeng Zhai, Xiaoxia Shi, Xin Liu, Shufeng Yang

**Affiliations:** ^1^Department of Microbiology, College of Basic Medical Sciences, Dalian Medical University, Dalian, China; ^2^Department of Biochemistry and Molecular Biology, College of Basic Medical Sciences, Dalian Medical University, Dalian, China; ^3^Department of Clinical Laboratory, Dalian Third People’s Hospital, Dalian, China; ^4^Department of Clinical Laboratory, Dalian Municipal Women and Children’s Medical Center, Dalian, China; ^5^Department of Occupational and Environmental Health, Dalian Medical University, Dalian, China

**Keywords:** bacille Calmette-Guérin, glycoprotein, O-mannosylated protein, vaccine, oligosaccharide chains

## Abstract

Bacterial glycoproteins have been investigated as vaccine candidates as well as diagnostic biomarkers. However, they are poorly understood in *Mycobacterium bovis* strain bacille Calmette-Guérin (BCG), a non-pathogenic model of *Mycobacterium tuberculosis*. To understand the roles of secreted O-mannosylated glycoproteins in BCG, we conducted a ConA lectin-affinity chromatography and mass spectra analysis to identify O-mannosylated proteins in BCG culture filtrate. Subsequent screening of antigens was performed using polyclonal antibodies obtained from a BCG-immunized mouse, with 15 endogenous O-mannosylated proteins eventually identified. Of these, BCG_0470 and BCG_0980 (PstS3) were revealed as the immunodominant antigens. To examine the protective effects of the antigens, recombinant antigens proteins were first expressed in *Mycobacterium smegmatis* and *Escherichia coli*, with the purified proteins then used to boost BCG primed-mice. Overall, the treated mice showed a greater delayed-type hypersensitivity response *in vivo*, as well as stronger Th1 responses, including higher level of IFN-γ, TNF-α, and specific-IgG. Therefore, mannosylated proteins BCG_0470 and BCG_0980 effectively amplified the immune responses induced by BCG in mice. Together, our results suggest that the oligosaccharide chains containing mannose are the antigenic determinants of glycoproteins, providing key insight for future vaccine optimization and design.

## Introduction

Tuberculosis (TB), caused by *Mycobacterium tuberculosis* (*M. tuberculosis*), remains a major threat to public health, with ever increasing morbidity and mortality rate worldwide (WHO, 2019). As reported by the World Health Organization in 2019, *M. tuberculosis* causes active TB disease in 10.0 million individuals each year, amongst whom approximately 1.5 million will die (WHO, 2019). Current TB health interventions include treatment of latent TB infection and childhood immunization using the bacille Calmette-Guérin (BCG) vaccine (Martinez et al., 2017; WHO, 2019). However, latent TB infection prevention services are often not accessible to eligible patients, BCG vaccination coverage generally achieves a rate of at least 90% (WHO, 2019). Thus, the efficient prevention resulting from the vaccine confirms its importance in fight against TB.

BCG, an attenuated *Mycobacterium bovis* strain developed as a vaccine by Calmette and Guérin in 1921, is the only licensed TB vaccine approved by the World Health Organization. Nowadays, BCG still is widely applicable in epidemic regions, especially for the prevention of extrapulmonary TB in infants (Toida, 2000; WHO, 2019). Although its efficacy is varied, nearly 100 years of BCG use confirms that this living bacilli alone inducessubstantial immune protection against TB (Pym et al., 2003; Nieuwenhuizen and Kaufmann, 2018). It is thought that the alive bacilli secret protective antigens once injected, promoting T-lymphocyte based immune responses and eliciting humoral immunity against TB (Horn et al., 1999; Pym et al., 2003). Therefore, identifying and characterizing the most immunogenic and efficient antigens in the BCG vaccine may help to completely eliminate TB infection.

Currently, many protein-based subunit vaccines have been developed based on highly immunodominant antigens secreted by the replicating *M. tuberculosis* or latency-associated proteins expressed by persistent bacilli (Kebriaei et al., 2016; Nandakumar et al., 2016; Khademi et al., 2018). A large number of secreted mycobacterial proteins have also been investigated as diagnostic biomarkers and vaccine candidates (Wolfe et al., 2010; Facciuolo and Mutharia, 2014). Some secreted *M. tuberculosis* proteins including alanine and proline-rich protein (Apa/Rv1860), Rv0934 (PstS-1), Rv3763 (LpqH), Rv1887, and Rv1096, are reportedly modified by O-linked mannosylation, the most common type of the post-translational modifications (Gonzalez-Zamorano et al., 2009; Sanchez et al., 2012; Nandakumar et al., 2013; Smith et al., 2014). Homologous glycoproteins in various other mycobacteria show structural diversity in their glycosyl moieties. For example, native Apa protein, secreted by *M. tuberculosis*, *M. bovis*, and BCG, contain one to nine mannose residues, while recombinant Apa expressed by *Mycobacterium smegmatis* contains seven to nine mannose residues. Interestingly, Apa from *Escherichia coli* does not contain mannose (Horn et al., 1999). Differences in mannosylation patterns are related to the T-cell antigenicity of Apa (Horn et al., 1999; Nandakumar et al., 2013), with mannosylated Apa proteins eliciting more robust lymphoproliferation response than those without mannose modification (Horn et al., 1999). Additionally, a key enzyme involved in the modification process, protein mannosyl transferase (Pmt, Rv1002c), is crucial for the virulence of *M. tuberculosis* (Liu et al., 2013). Together, these findings demonstrated the antigenic significance of mannosylated proteins in mycobacteria, and suggested mannose linked to mycobacterial proteins represents a potential antigenic determinant.

However, the significance of mannosylation in glycoproteins of BCG and the role of oligosaccharide chains linked to O-glycoproteins are poorly understood. To examine the mannosylated proteins of BCG, and understand their roles in eliciting an immune responses in the host, we carried out lectin affinity chromatography assays followed by mass spectra (MS)-based identification. The findings of this study will help us to understand the importance of mannosylated proteins and oligosaccharide chains containing mannose residues of BCG, and reveal the effective antigens for use in BCG vaccines.

## Materials and Methods

### Bacteria and Culture Conditions

Bacille Calmette-Guérin G Danish strain was reactivated on Middlebrook 7H10 agar (BD, Franklin Lakes, NJ, United States) supplemented with 10% OADC (oleic albumin dextrose catalase) at 37°C for 20 days. A single colony was picked up and inoculated in 5 ml of Proskauer and Beck modified synthetic medium (Gonzalez-Zamorano et al., 2009) and cultured to mid-log phase at 37°C. This starter culture was transferred into 200 ml of fresh Proskauer and Beck modified synthetic medium, and incubated without shaking until surface pellicles were visible. Bacterial cells were then removed by filtration using a 0.22-μm polyethersulfone filter, leaving only the culture filtrate. Proteins were precipitated by incubation with ammonium sulfate at 4°C overnight, and then collected by centrifugation at 10,300 × *g* for 30 min at 4°C. Protein pellets were resuspended in distilled water and dialyzed completely against distilled water at 4°C for 72 h. The dialyzed proteins were lyophilized and stored at −80°C.

### Animal Care

This study was carried out in accordance with the principles of the Basel Declaration and the Dalian Medical University recommendations for laboratory animals. The protocol was approved by the Animal Ethics Committee of Dalian Medical University.

### ConA-Affinity Chromatography

A 1 ml ConA-agarose column (Sigma-Aldrich, Stainheim, Sweden) was prewashed with five column volumes of wash solution (1 M NaCl, 5 mM MgCl_2_, 5 mM MnCl_2_, and 5 mM CaCl_2_) and the resin equilibrated with equilibrating buffer (20 mM Tris, 0.5 M NaCl, pH 7.4). Protein solution containing 2.0 mg of total protein from CF (1 mg/ml, free of particulates) was then loaded onto column and washed with equilibration buffer until the eluent solution was free of protein. The target proteins were eluted with solution containing 200 mM methyl-D-glucopyranoside (Sigma-Aldrich, Stainheim, Sweden). Samples were dialyzed, lyophilized and stored at −80°C until use.

### One-Dimensional and Bidimensional Gel Electrophoresis and Western Blot Analysis

Purified glycoproteins were subjected to 12% sodium dodecyl sulfate-polyacrylamide gel electrophoresis (SDS-PAGE) using a vertical electrophoresis apparatus. Gels were silver-stained according to the manufacturer’s instructions. In addition, bidimensional gel electrophoresis (2D-DIGE) was performed as described previously (Chen et al., 2019). Briefly, samples were processed using a Bio-Rad clean-up kit as per the manufacturer’s instructions (Hercules, CA, United States). The protein pellets were then dissolved in 50 μl of isoelectric focusing buffer (30 mM Tris, pH 8.0, 7 M urea, 2 M thiourea, 2% CHAPS). Soluble mannoproteins (30 μg) were separated in the first dimension using Immobiline DryStrips (11 cm, pH 4–7, Bio-Rad) and in the second dimension using 12% SDS-PAGE gels. The gels were stained using a ProteoSilver Plus SilverStain Kit (Sigma-Aldrich, MO, United States).

The proteins were then transferred from the SDS-PAGE gels onto polyvinylidene fluoride (PVDF) membrane (Millipore, Prod, pore size 0.45 μm) using Trans-Blot machine (Bio-Rad, Hercules, CA). The parameters were setting at 110 V and 35 min. The membrane was blocked with 2% (w/v) bovine serum albumin (BSA) in phosphate-buffered saline (PBS) and then incubated with 10 μg/ml biotinylated ConA (Sigma-Aldrich, Stainheim, Sweden) for 2 h at room temperature. The membrane was then incubated with streptavidin-peroxidase (1:5000, Sigma-Aldrich, St. Louis, MO, United States) solution as per the manufacturer’s instructions. The target bands were visualized using enhanced chemiluminescence reagents (Millipore, Burlington, MA, United States).

### MS Analysis

#### Matrix-Assisted Laser Desorption/Ionization Time-of-Flight (MALDI-TOF)

Selected silver-stained proteins were manually excised from the 2D-PAGE gels for MS-based analysis. Gel digestion and MS analysis (5800 MALDI-TOF, AB SCIEX, Foster City, CA, United States) were performed as described previously (Yang et al., 2018). Data were acquired using Mascot 2.2 software (Matrix Science)^1^.

#### Liquid Chromatography Tandem Mass Spectrometry (LC-MS/MS)

Whole glycoproteins digested with trypsin were also examined using a Q Exactive Mass Spectrometer (Thermo Fisher Scientific, Waltham, MA, United States) coupled with an Easy nLC liquid chromatography system (Thermo Fisher Scientific). LC was operated by a C18 reverse phase trap column (Scientific Acclaim PepMap100, 100 μm × 2 cm, nanoViper, Thermo Fisher Scientific) connected to a C18 reversed-phase analytical column Easy Column, 10 cm long, 75 μm inner diameter, 3 μm resin, Thermo Fisher Scientific) in 0.1% formic acid and separated with a linear gradient of buffer (acetonitrile and 0.1% formic acid). The flow rate was at 300 ml/min and the system was controlled using IntelliFlow technology. The MS system was operated in positive ion mode. The optimal source/gas parameters were as follows: automatic gain control target, 3e6; maximum inject time, 10 ms. The dynamic exclusion duration was 40.0 s and the normalized collision energy was 30 eV. The survey scan for higher-energy collisional dissociation (HCD) fragmentation was set at 300–1800 m/z, with HCD spectra set to 17,500 at m/z 200, and an isolation width was of 2 m/z. The instrument was run with an enabled mode of peptide recognition.

### Preparation of Murine Anti-Serum Against BCG

BALB/c female mice (8 mice per group) were subcutaneously immunized with 5 × 10^5^ colony-forming units (CFU) of log-phase BCG in normal saline). The control group was injected with equal volume of normal saline. At 3 weeks post-inoculation, whole blood were collected and centrifuged with 1,000 × *g* for 10 min. The sera were then collected for the detection of mannosylated antigens.

### Detection of Mannosylated Antigens

Glycoproteins separated by SDS-PAGE and 2D-PAGE were transferred onto PVDF membrane as described. After blocking with 5% BSA (w/v) in PBS, the membranes were blotted with the prepared polyclonal antibodies (1:1,000) overnight at 4°C. Following incubation, the membranes were washed three times with PBS and then incubated with the secondary antibody (1:5000, rabbit anti-rat IgG, HRP conjugated; Sigma-Aldrich, MO, United States) for 1.5 h at room temperature. Protein bands and spots were visualized using enhanced chemiluminescence. Target bands and spots were then excised manually and stripped using Restore Western Blot Stripping Buffer (Thermo Fisher Scientific, United States) at 37°C for 40 min to remove affinity-bound primary and secondary antibodies. After washing with tris-buffered saline with 0.1% Tween 20, the target proteins were identified by LC-MS/MS as described previously.

### Expression, Purification, and Treatment of the Identified BCG Antigens, Including BCG_0470 and BCG_0980

The *BCG_0470* and *BCG_0980* coding sequences were amplified from BCG genomic DNA using *Pfu* High Fidelity DNA Polymerase (Invitrogen, Carlsbad, CA, United States) and the following primers: BCG_0470-F (5′-CATATGGTGCTGGTT ACAGTGGGCTC-3′, *Nde*I site is underlined), paring with BCG_0470-R (5′-AAGCTTTCAGCCGGTCACCACGAC-3′, *Hin*dIII site is underlined); and BCG_0980-F (5′-CA TATGTTGAAACTCAACCGATTTGG-3′, *Nde*I site is underlined), pairing with BCG_0980-R (5′-AAGCTT TCAGGCGATCGCGTTGACCG-3′, *Hin*dIII site is underlined). The amplified products were then ligated into the pJET (Thermo Fisher Scientific, Lithuania) cloning vectors. Following sequence confirmation, the amplicons were excised from the vectors using the appropriate restriction enzymes and ligated into pCold II (Takara, Dalian, China),which carries a hexa-histidine tag at the C-terminus and a cold start promoter, generating recombinant the expression vectors pCold II-*BCG_0470* and pCold II-*BCG_0980*. For expression, the plasmids were transformed into *E. coli* BL21 (DE3). In addition, recombinant plasmids pVV2-*BCG_0470* and pVV2-*BCG_098* were constructed to allow expression of the two proteins in *M. smegmatis*. The vectors were transformed into *M. smegmatis* by electroporation as described previously (Yang et al., 2014). Cell lysates were then prepared to purify recombinant BCG_0470 and BCG_0980 as described previously (Yang et al., 2018). The recombinant proteins were assessed by 12% SDS-PAGE and further confirmed by western blot using anti-His monoclonal antibody (Sigma-Aldrich, St. Louis, MO, United States) or ConA lectin (Sigma-Aldrich, St. Louis, MO, United States) as a probe. In addition, the recombinant mannosylated proteins were treated with α-mannosidase (Sigma-Aldrich, St. Louis, MO, United States) as per the manufacturer’s instructions. Mannosidase-treated proteins were collected using magnetic agarose beads as described previously (Chen et al., 2019).

### Immunization of BCG-Primed Mice Using the Recombinant Antigens

**FIGURE 1:**
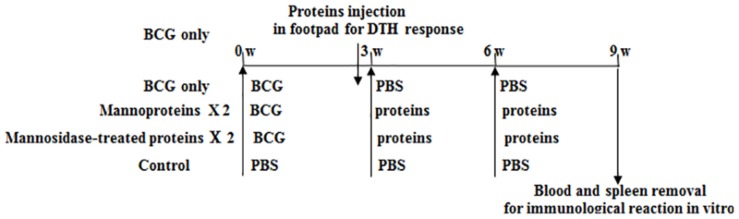
Schematic diagram of immunization. Female BALB/c mice were primed with 1 × 10^5^ CFU of BCG by intravenous injection, then boost twice at 3 weeks intervals with recombinant proteins (BCG_0980 and BCG_0470) in presence or absence of mannose modification.

Female BALB/c mice (five mice per group) were primed with 1 × 10^5^CFU of BCG by intravenous injection as shown in Figure 1. At 3 weeks post-inoculation, 5 μg of the recombinant proteins (with or without mannose modification) were subcutaneously injected into the BCG-primed mice, followed by a second twice dose at 6 weeks post-inoculation. An additional group of mice were injected with an equal volume of PBS as a negative control. At 3 weeks after the final booster, the animals were sacrificed to evaluate immune responses *in vitro*.

### Evaluation of Delayed-Type Hypersensitivity (DTH) Responses

The DTH responses of mice to recombinant protein with or without mannose modification were evaluated in mice inoculated with BCG only (no recombinant protein booster) as previously described (Tong et al., 2018). Briefly, as shown in Figure 1, at 2 weeks 4 days post-BCG immunization, 5 μg of recombinant protein (four groups: BCG_0470, mannosidase-treated BCG_0470, BCG_0980, and mannosidase-treated BCG_0980) in 40 μl of PBS were injected into the left hind footpad, while the same volume of PBS was injected into the right hind footpad as a control. Differences in footpad swelling between the protein-challenged and PBS-injected feet were measured at 24, 48, and 72 h post-protein injection to assess protein-specific DTH responses. In addition, at 48 h post-protein injection, three mice of every group were then sacrificed and equal amounts of the left hind footpads were collected and homogenized in 1 ml of protein extraction buffer with protease inhibitor cocktail (Thermo Fisher Scientific). The homogenates were centrifuged at 10,000 × *g* for 10 min to collect the supernatants, which were then tested to determine the levels of various cytokines, including IFN-γ, IL-4, and TNF-α, using an enzyme-linked immunosorbent assay (ELISA) kit (Abcam, Hong Kong, China) as per manufacturer’s instruction (Aguilar et al., 2007).

### Detection of Antigen Specific Antibodies Using ELISA

Whole blood from sacrificed mice was centrifuged at 1,000 × *g* for 10 min at 4°C, with the resulting serum collected and immediately used to assess the specificity of antibodies against their corresponding antigens. Briefly, 150 μl of each (2 μg/ml) recombinant antigen was incubated overnight at 4°C in a 96-well plate. After blocking with 3% BSA in PBS, the plate was incubated with diluted (1:1,000) murine serum for 2 h at 37°C, before being incubated with horseradish peroxidase-conjugated anti-mouse IgG (1:5000 dilution) at room temperature for 1 h. Tetramethylbenzidine was added to each well for color development, which was then stopped with 2 M sulfuric acid. The optical density of each well was then measured at 450 nm.

### Detection of Cytokine Secretion in Mouse Splenocytes by ELISA

Spleens from the sacrificed mice were aseptically removed, and the single-cell suspensions were prepared as reported previously (Rao et al., 2017). The freshly isolated splenocytes were resuspended in RPMI 1640 medium (Gibco, Suzhou, China) enriched with 10% fetal calf serum and antibiotics (penicillin and streptomycin) at concentration of 1 × 10^6^ cells/ml. The cells were then seeded in a 96-well sterilized plate and stimulated with 2 μg/ml antigen mixture (BCG_0980 and BCG_0470) *in vitro* for 72 h at 37°C with 5% CO_2_. The plate was then centrifuged at 1,600 × *g* for 10 min to collect supernatant, which was subsequently used to measure the levels of various cytokines including TNF-α, IFN-γ, and IL-2, using ELISA kits (Abcam, Hong Kong, China).

### Statistical Analysis

All the data were are presented as mean ± SEM from at least three independent experiments. Statistical analyses were performed using a Student’s unpaired *t*-test or one-way ANOVA. Unless stated, *p* < 0.05 was considered statistically significant.

## Results

### Recognition of the Mannosylated Proteins in BCG

**FIGURE 2:**
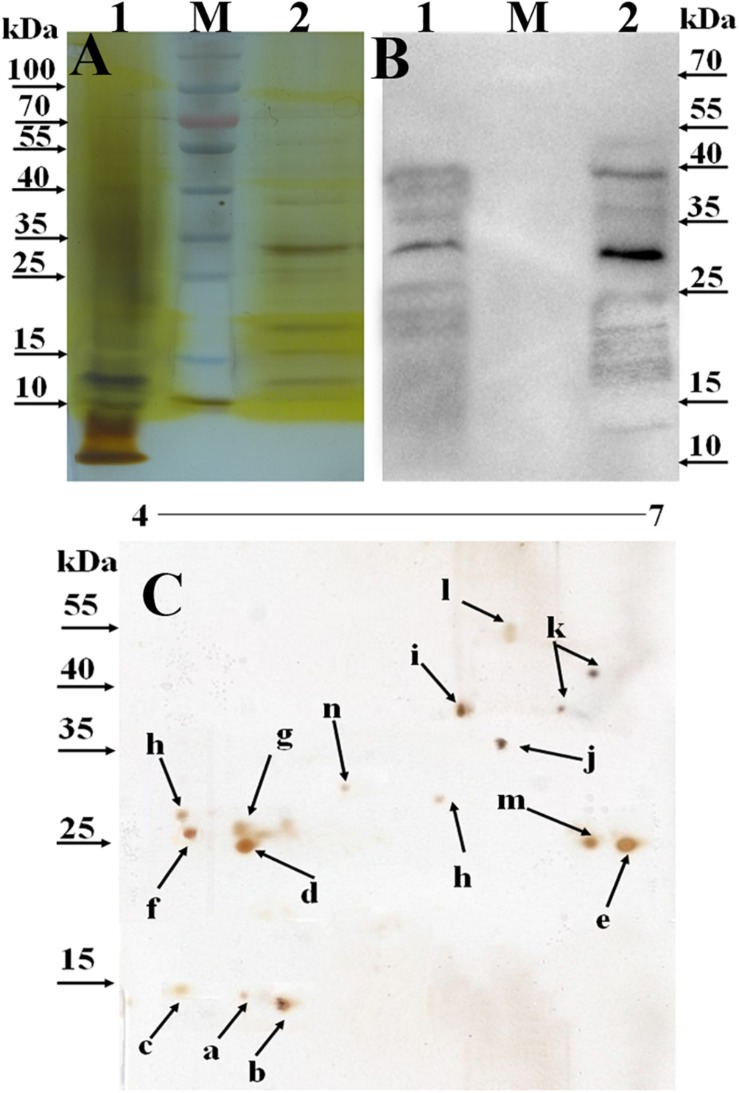
Identification of secreted O-mannosylated proteins in BCG using SDS-PAGE, 2D-PAGE, and Western blot. M, PageRuler prestained protein ladder (Thermo Scientific, Lithuania). 1, the total secreted proteins precipitated by (NH4)_2_SO_4_; 2, ConA-dragged O-mannosylated proteins from BCG CF. (A) SDS-PAGE analysis of the secreted O-mannosylated proteins in BCG. The gel was visualized using sliver staining kit. (B) Western blot analysis of the secreted O-mannosylated proteins in BCG. The membranes were blotted with ConA, and visualized using ECL. (C) 2D-PAGE analysis of the secreted O-mannosylated proteins in BCG. The gel was visualized using sliver staining kit. a–n, the spots selected for MALDI-TOF identification. Five microgram proteins were loaded.

To identify O-mannosylated proteins in the BCG CF, ConA-lectin affinity chromatography was used. The glycoproteins were eluted using the solution containing a high concentration of methyl-α-mannopyranoside. SDS-PAGE and 2D-PAGE analyses were utilized to separate the purified glycoproteins. Bands and spots corresponding to the glycoproteins on the SDS-PAGE (Figure 2A) and 2D-PAGE (Figure 2C) were visualized by silver-staining. In total, 9 protein bands with a molecular mass of 10–70 kDa were observed on the SDS-PAGE gel. While 14 proteins spots (Figure 2Ca–n) were selected from the 2D-PAGE gel. Western blot analysis confirmed the binding activity of the observed proteins with ConA. As shown in Figure 2B, the majority of mannoproteins present in the CF were identified using ConA-lectin.

### Identification of the Mannosylated Proteins in BCG

**FIGURE 3:**
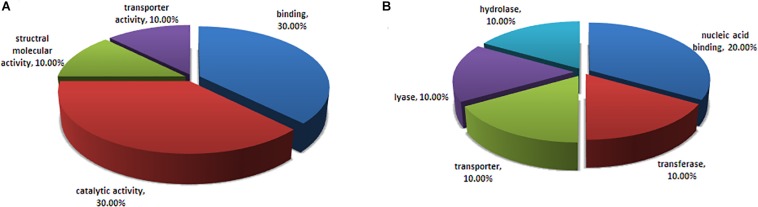
Classification analysis of the 15 identified glycoproteins in BCG using the online PANTHER database. Representative pie charts display the enrichment percentage of proteins. **(A)** Proteins are classified based on molecular functions. **(B)** Proteins are categorized based on protein classes.

Fourteen visible spots on the silver-stained 2D-PAGE gel (Figure 2Ca–n) were excised for MALDI-TOF-based identification, while whole glycoproteins were characterized by LC-MS/MSanalysis. Overall, 15 glycoproteins were identified (Table 1), amongst which BCG_2967c (LppX), BCG_0980 (PstS3) and BCG_1472c (LprG) were shown to be lipoproteins with potential sites for type II signal peptidase binding. Further, BCG_3277c, BCG_3252, BCG_1154, and BCG_1564 were predicted to be involved in degradation and metabolism of nutrients, BCG_1896 (Apa) was associated with cell adhesion, and BCG_RN06_2923 and BCG_1237c were predicted to play a role in bacterial growth. All identified proteins were predicted to have potential signal peptides. Most of them were evaluated to possess between 1 and 23 potential O-glycosylated sites. Result of the classification analysis of 15 identified glycoproteins based on molecular function and protein class are shown in Figure 3.

**TABLE 1:** Profiles of the identified mannosylated proteins secreted by BCG.

ID	BCG gene	Access No.*	Protein name	Protein MW (D)	Matched peptides	Sequence coverage %	NetOglyc hits^#^	Predicted Lpp^&^	*M. bovis* ortholog	*M. tuberculosis* ortholog	Identified MS methods^1/2^
a	0086	A1KEM3	30S ribosomal protein S18 rpsR1	9543.1	1	8.33	1		Mb0056	Rv0055	^1^
b	1237c	A0A0H3MC91	SA5K secreted antigen	10881.2	2	9.09			Mb1207c	Rv1174c	^1^
c	RN06_ 2923	A0A109S8K1	Resuscitation-promoting factor RpfD	15678.6	1	3.08	3		Mb2410c	Rv2389c	^1^
d	2967c	A0A0K2HZQ7	Putative phthiocerol dimycocerosate transporter LppX	24139.9	2	8.58	6	yes	Mb2970c	Rv2945c	^1^
e	1472c	A0A0H3M3S8	Lipoarabinomannan carrier protein LprG	24547.5	1	2.97	1	yes	Mb1446c	Rv1411c	^1^
f	0470	A0A0H3M1J1	Putative tuberculin related peptide	16865.3	1	8.39	11		Mb0439	Rv0431	^1^
g	3419c	A0A0H3MEK7	Probable transposase	26630.2	1	9.08	9		Mb3382c	Rv3349c	^1^
h	1154	A0A0H3MC31	Possible acyl-[acyl-carrier protein] desaturase (desA2)	31359.1	1	3.64	2		Mb1124	Rv1094	^1^
i	0980	A0A0H3MBK5	Phosphate-binding protein3 (PstS3)	37952.7	4	7.57	7	yes	Mb0951	Rv0928	^1^
j	1156	A0A0H3M373	Possible glycosyl hydrolase	31099.1	2	2.69	3		Mb1126	Rv1096	^1^
k	1896	P80069	Alanine and proline-rich secreted protein Apa	32686.3	6	9.23	16		Mb1891	Rv1860	^1^
l	3277c	A1KNQ0	Adenosylhomocysteinase (ahcY)	54323.3	1	2.82	4		Mb3276c	Rv3248c	^1^
m	3652c	A0A0H3MBJ4	Probable conserved membrane protein	27001.5	1	4.94	14		Mb3618c	Rv3587c	^1^
n	3955c	A0A0H3MFZ4	Uncharacterized protein	29993.6	1	2.39	23		Mb3928c/Mb3829c	Rv3899c	^1^
	3252	A0A0G2Q9I2	Possible short-chain dehydrogenase/reductase	29814.1	1	3.55	0		Mb3251	Rv3224	^2^

*^1^Identified by both MALDI-TOF and LC-MS/MS. ^2^only identified by LC-MS/MS. ^∗^UniprotKB access number. ^#^According to NetOglyc predictions (http://www.cbs.dtu.dk/services/NetOGlyc/, NetOGlyc 4.0 Server). ^&^According to the predictions of lipoproteins (https://services.healthtech.dtu.dk/service.php?LipoP, LipoP1.0 Server).*

### Confirmation of BCG_0470 and BCG_0980 as Immunodominant Antigens

**FIGURE 4:**
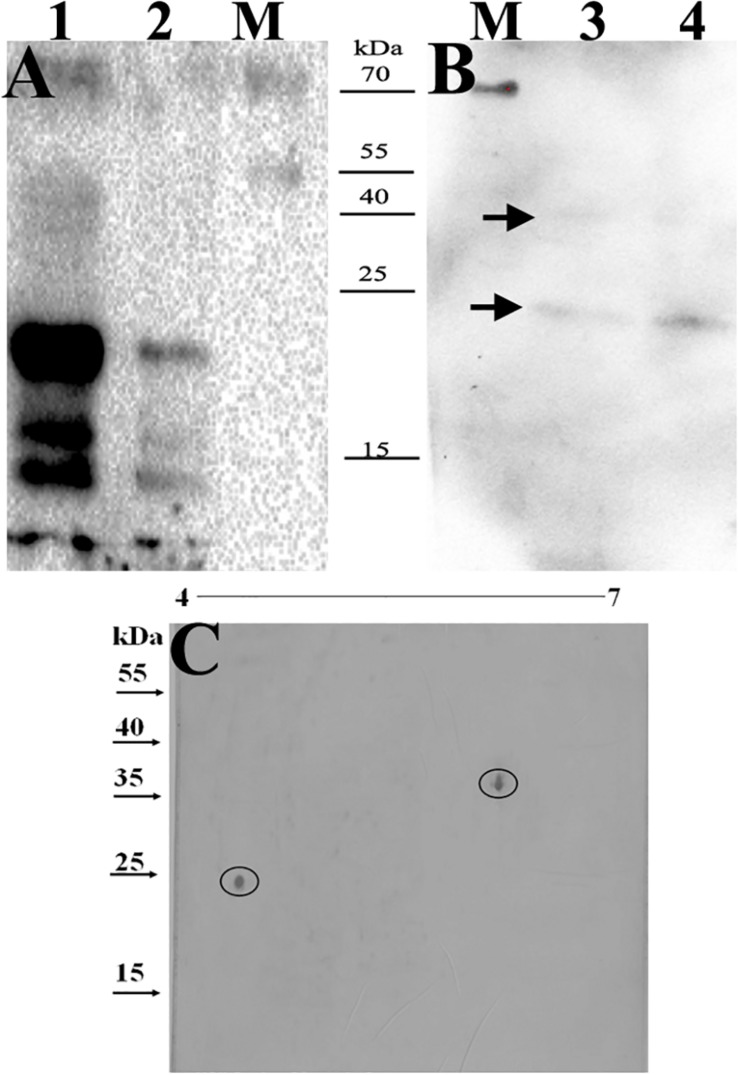
Identification of the immunodominant glyco-antigens by BCG ploycolonal antibody using immunoblotting. 5μg proteins were loaded. M, PageRuler prestained protein ladder (Thermo Scientific, Lithuania). **(A,B)** the samples were separated by SDS-PAGE. 1, whole BCG cell-lysates; 2, crude protein extracts precipitated by the addition of (NH4)_2_SO_4_; 3 and 4, mannosylated proteins purified from BCG CF. Arrows indicated two targeting bands with molecular masses of approximately 38 kDs and 25 kDa, respectively. **(C)** Samples were separated by 2D-PAGE analysis. Circles indicate two targeting spots with molecular masses of approximately 38 kDs and 25 kDa, respectively.

Based on the possible involvement of the identified glycoproteins in immune responses, BCG ploycolonal antibody was used as a probe to screen potential immunogenic antigens (v:v, 1:1,000). Figure 4A shows that at least seven bands were detected in the whole BCG cell lysates, while four bands were detected in crude protein extracts, which were precipitated by the addition of ammonium sulfate. Among the antigens, two proteins with molecular masses of approximately 25 and 38 kDa, respectively were detected. In addition, the polyclonal antisera showed cross-reactivity with BCG between whole BCG cell lysates and ConA-dragged proteins (Figure 4A), and bands with molecular masses approximately 25 kDa and 38 kDa were also detected among the ConA-dragged proteins (Figure 4B). Because bands observed on SDS-PAGE gels can comprise more than one protein, immunoblotting of ConA-dragged proteins separated by 2D-PAGE analysis was performed. Results confirmed the presence of only two target spots (Figure 4C).

**FIGURE 5:**
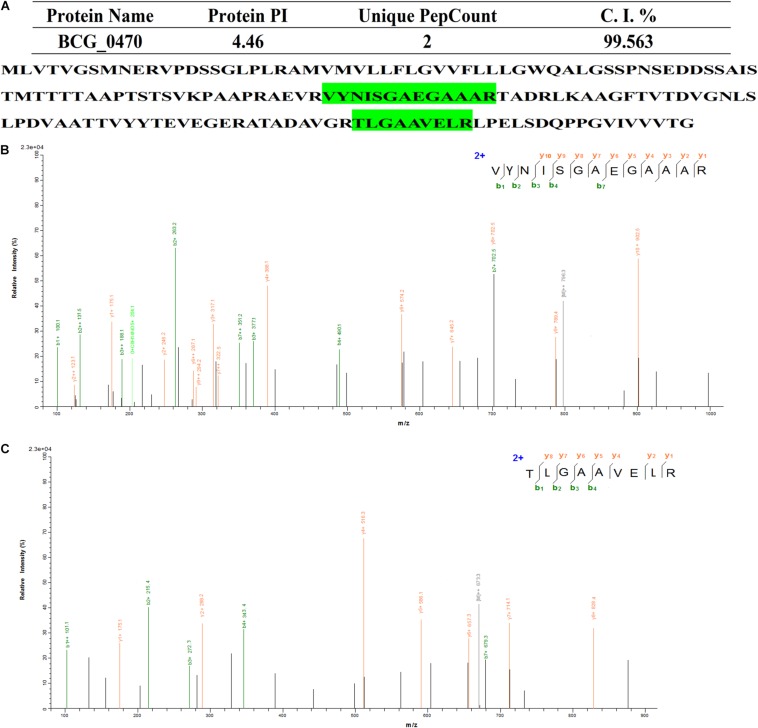
Identification of BCG_0470 via LC-MS/MS-based analysis. **(A)** Profiles of BCG_0470. **(B,C)** Tandem mass spectrometry (MS/MS) spectrum of the trypsin-digested BCG_0470. **(B)** Peptide fragment ions with m/z 929.5441: sequence TLGAAVELR. **(C)** Peptide fragment ions with m/z 1278.6677: sequence VYNISGAEGAAAR.

**FIGURE 6:**
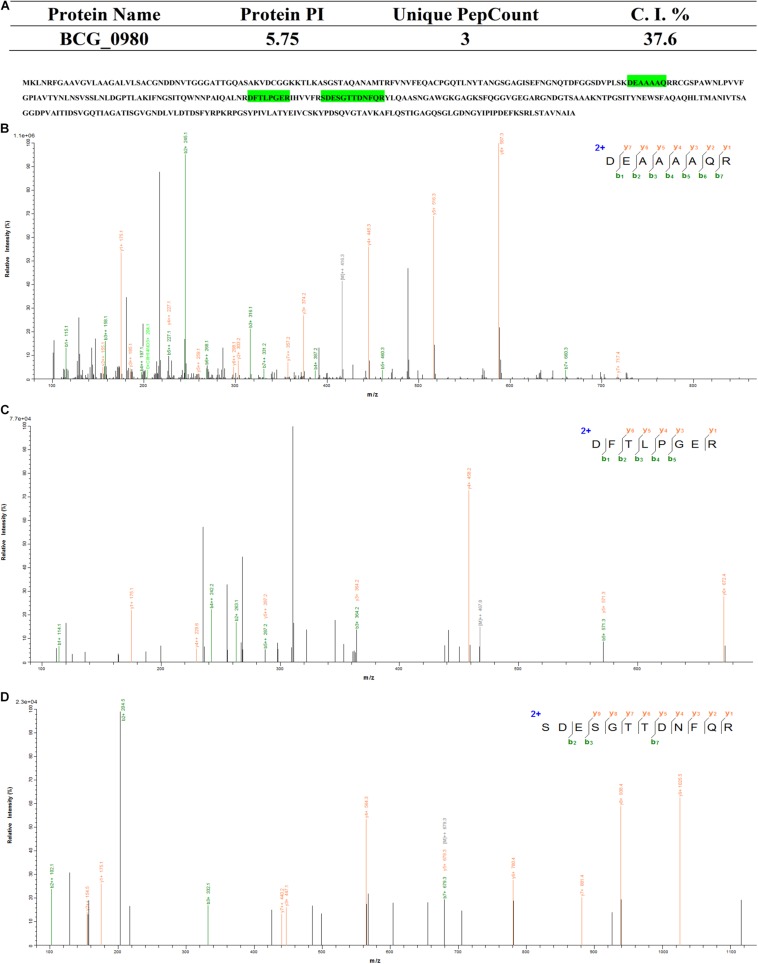
Identification of BCG_0470 via LC-MS/MS-based analysis. **(A)** Profiles of BCG_0980. **(B–D)** Tandem mass spectrometry (MS/MS) spectrum of the trypsin-digested BCG_0980. **(B)** Peptide fragment ions with m/z 831.3955: sequence DEAAAAQR. **(C)** Peptide fragment ions with m/z 934.46287: sequence DFTLPGER**. (D)** Peptide fragment ions with m/z1356.56624: sequence SDESGTTDNFQR.

We then further characterized the 15 identified glycoproteins with immunogenic activities, we analyzed the 15 identified proteins. As a consequence, Glycoproteins BCG_2967, BCG_1472c, BCG_0470, BCG_3419c, BCG_1154, and BCG_3652c (spot IDs d, e, f, g, h, and m, respectively, in Figure 2C) were all found to be ∼25kDa, while BCG_0980 was ∼38 kDa. To further confirm the targeting glycoproteins detected in Figures 4B,C, the reactive spots were excised and stripped for LC-MS/MS-based analysis. MS analysis confirmed that the target proteins were BCG_0470 (spot ID f in Figure 2C, 25 kDa) and BCG_0980 (spot ID i in Figure 2C, 38 kDa). The MS profiles of BCG_0470 and BCG_0980 are shown in Figures 5, 6.

### Characterization of Mannosylated BCG_0470 and BCG_0980

**FIGURE 7:**
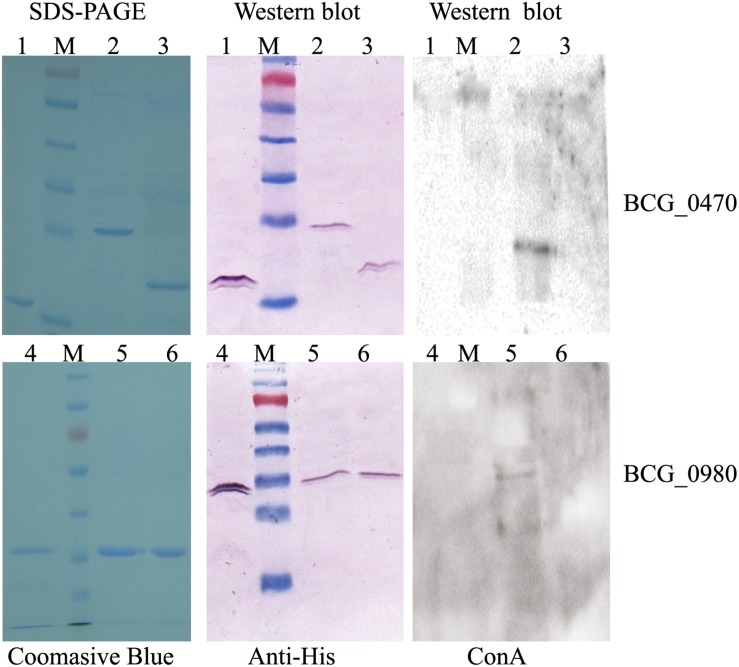
Characterization of BCG_0470 and BCG_0980 as mannosylated proteins. 1, Recombinant BCG_0470 from *E. coli*. 2, Recombinant BCG_0470 from *M. smegmatis*. 3, Recombinant BCG_0470 from *M. smegmatis* treated by α-mannosidase. 4, Recombinant BCG_0980 from *E. coli*. 5, Recombinant BCG_0980 from *M. smegmatis*. 6, Recombinant BCG_0980 from *M. smegmatis* treated by α-mannosidase.

To further characterize the mannosylation of BCG_0470 and BCG_0980, the proteins were expressed in both *E. coli* and *M. smegmatis*, respectively. Previous studies have shown that expressed proteins are non-glycosylated in *E. coli*, but are glycosylated in *M. smegmatis* (Coddeville et al., 2012). As shown in Figure 7, the soluble proteins were successfully expressed in both *E. coli* and *M. smegmatis* in this study. The molecular mass of BCG_0470 was much higher when expressed in *M. smegmatis* (∼25 kDa) than in *E. coli* (∼ 18 kDa). Non-glycosylated BCG_0470 has a predicted molecular mass of 18 kDa, which agreed with the mass of protein expressed in *E. coli*. In addition, western blot analysis showed that BCG_0470 expressed in *M. smegmatis* reacted strongly with ConA-lectin (Figure 7). In comparison, SDS-PAGE and western blot analysis showed that the molecular mass of recombinant BCG_0980 was similar in both *E. coli* and *M. smegmatis*, and was consistent with the predicted molecular mass ∼38 kDa. Western blot analysis blotted with ConA showed a weak band corresponding to BCG_0980 when expressed in *M. smegmatis*. Therefore, these findings suggested that BCG_0470 is highly mannosylated while BCG_0980 is mannosylated to a lesser extent.

To examine mannose linkage in BCG_0470 and BCG_0980, α-mannosidase, which hydrolyzes the terminal non-reducing α-D-mannose residues of glycoproteins, was used to digest the recombinant BCG_0470 and BCG_0980 proteins expressed in *M. smegmatis*. Western blot analysis showed no ConA binding, suggesting complete loss of mannose residues from the α-mannosidase-digested BCG_0470 and BCG_0980 proteins. Thus, the examined mannosylated proteins, including BCG_0470 and BCG_0980, are likely to contain a terminal non-reducing α-D-mannose.

### Confirmation T-Cell Activator by BCG_0470 and BCG_0980 via DTH Responses *in vivo*

**FIGURE 8:**
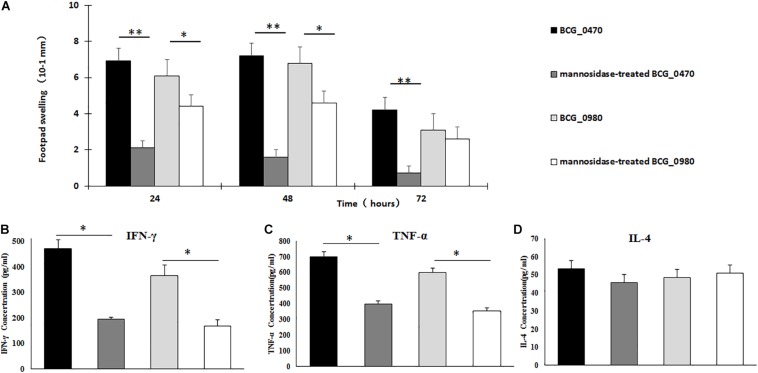
Detection of T-cell activity in the BCG-sensitized BALB/c mice via DTH responses. **(A)** Measurement of swelling in protein-challenged footpad. Both mannosylated BCG_470 and BCG_0980 could elicit increased swelling-responses than non-mannosylated proteins, especially in BCG_0470 (*t*-test, *p < 0.05, **p < 0.01). **(B–D)** Cytokines including IFN-γ **(B)**, TNF-α **(C)**, and IL-4 **(D)** in 48 h post protein-challenged footpad. Both mannosylated BCG_0470 and BCG_0980 could induce higher level of IFN-γ, IFN-α than non-mannosylated proteins. The level of IL-4 showed no difference between the paired groups (*t-*test, *p < 0.05, **p < 0.01).

Recombinant BCG_0470 and BCG_0980 proteins with or without mannose modification were injected intradermally into the hind footpads of BCG-sensitized BALB/c mice. Footpad swelling was then recorded at 24 h intervals. As shown in Figure 8A, increased swelling response was observed in mice injected with the mannosylated proteins compared with those injected with non-mannosylated proteins. Additionally, a significant difference in footpad swelling was observed at 24 h and 48 h post-injection time points, especially in BCG_0470-injected mice. To further determine whether the footpad swelling resulted from DTH or simple tissue edema, three mice of every group were sacrificed at 48 h post-injection time point, the levels of DTH-associated cytokines in the protein-injected footpads were assessed. As shown in Figures 8B,C, higher levels of IFN-γ and TNF-α were observed in the mannoprotein-injected footpads compared with those injected with mannosidase-treated proteins, but the level of IL-4 showed no difference between the paired groups (Figure 8D). Thus, footpad swelling was mainly attributed to DTH-associated Th1 cytokines, which attract cells to the injection site. Additionally, analysis of the DTH response demonstrated the T-cell activity of mannosylated BCG_0470 and BCG_0980 in the BCG-sensitized BALB/c mice, suggesting that mannose residues play an important role in immune responses *in vivo*.

### Detection of BCG_0470- and BCG_0980-Induced Splenocyte Cytokines and Antigen-Specific IgG

**FIGURE 9:**
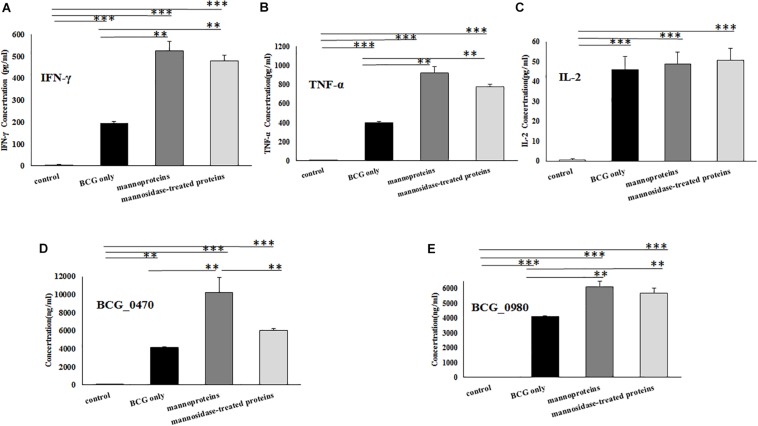
Detection the levels of splenocytes cytokines and serum antigenic-specific IgG using ELISA. **(A)** IFN-γ. **(B)**TNF-α. **(C)**IL-2. **(D)** BCG_0470-specific IgG. **(E)** BCG_0980-specific IgG. Mice were primed with BCG and received two boosts of BCG_0470 and BCG_0980 proteins with or without mannose modification. Differences between the groups were calculated by One Way ANOVA. Asterisks represented statistical difference (one-way ANOVA, *p < 0.05, **p < 0.01, ***p < 0.001).

BCG-primed BALB/c mice were subcutaneously injected at two separate time points with recombinant BCG_0470 and BCG_0980 proteins with or without mannose modification. At three weeks post final immunization, splenocytes were isolated for detection of cytokines including IFN-γ, TNF-α, and IL-2while the serum was collected and assayed to determine the levels of antigen-specific IgG. As shown in Figure 9, all immunized mice showed dramatically increased levels of cytokines and antigen-specific IgG compared with PBS-injected mice, confirming the antigenicity of BCG and two boosting proteins. In addition, compared with the mice only received BCG injection, mice treated with mannosylated or non-mannosylated protein booster injections showed increased levels of IFN-γ and TNF-α (Figures 9A,B). In comparison, no significant differences in IL-2 levels were detected between the paired groups (Figure 9C). Further, mice that received either mannosylated or non-mannosylated proteins boosts showed increased level of BCG_0980-specific IgG, compared with mice received BCG only (Figure 9E). Interestingly though, only mice treated with mannosylated protein boosters showed increased production of BCG_0470-specific antibody, with no significant increase in specific IgG antibody in non-mannosylated protein-treated mice compared with the BCG-only group (Figure 9D). Overall, these results suggested that BCG_0470 and BCG_0980 amplify the immune responses induced by BCG, and imply that the mannose oligosaccharide of BCG_0470 is indispensable for promoting high levels of BCG_0470-specific IgG.

## Discussion

Protein O-mannosylation process occurs in both eukaryotes and prokaryotes, including the important human pathogen *M. tuberculosis*, the etiological agent of TB (Espitia and Mancilla, 1989). O-mannosylated proteins play an important role in bacterial physiology and host-pathogen interactions (Liu et al., 2013; Alonso et al., 2017). Since the initial discovery of mycobacterial glycoproteins such as Apa, PstS1, and LpqH (Espitia and Mancilla, 1989), many studies have focused on this subclass of proteins. To date, more than 40 proteins secreted by *M. tuberculosis* have been identified as mannoproteins (Gonzalez-Zamorano et al., 2009; Smith et al., 2014), while Sec-dependent translocation has been shown to be required for the export of *M. tuberculosis* mannoproteins (VanderVen et al., 2005). In addition, the glycosylation sites and the structural linkage of O-linked glycoproteins have been reported (Horn et al., 1999; Smith et al., 2014; Alonso et al., 2017). In the current study, we identified 15 mannosylated proteins from BCG CF. The proteins had a wide range of predicted functions, including catalytic activity, solute binding, cell wall biosynthesis, and ABC transporters (Table 1 and Figure 3). Of the 15 identified glycoproteins, lipoprotein BCG_2967c (LppX) has been confirmed as a B-cell immunogenic protein expressed on the BCG cell surface (Lefevre et al., 2000). *M. tuberculosis* PstS3 is reportedly an excellent immunomodulator involved in both Th1 and Th17 responses (Palma et al., 2011), and is also a promising sera-diagnostic marker for latent tuberculosis infection (Baumann et al., 2013, 2015). BCG_1472c (LprG) is another important lipoprotein identified in this work. Its ortholog in *M. tuberculosis*, coded by *Rv1141c*, is described as a lipoarabinomannan carrier protein with Toll-like receptor 2 (TLR-2) agonist activity (Drage et al., 2010). LprG is also thought to be a virulence factor because a *M. tuberculosis Rv1141c* deletion mutant showed reduced virulence in a mouse model of infection (Gaur et al., 2014). In addition, LprG is the key target glycoprotein responsible for the impaired growth and attenuated virulence associated with *pmt* (*Rv1002c*) deletion in *M. tuberculosis* (Yang et al., 2014; Alonso et al., 2017). The impaired growth and virulence of the *pmt* mutant was also observed in the corresponding *Mycobacterium abscessus* mutant strain (Becker et al., 2017). Furthermore, secreted antigen SA5K (also called TB8.4 low molecular weight T-cell antigen), encoded by *BCGsa5k*, is associated with BCG intracellular adaption and survival (Bottai et al., 2006), while resuscitation-promoting factor RpfD, encoded by *RN06_2923* in BCG, demonstrates strong immunogenicity in many mycobacterial infection models (Romano et al., 2012). Taken together, these findings suggested that the O-mannosylated proteins identified in the current study were potentially antigenic, and could be used to promote the immune responses induced by BCG.

Polyclonal antisera from mycobacterial-infected patients or animals could be used to screen for the immunogenic proteins (Wieles et al., 1994; Facciuolo et al., 2013) (Tsibulkin et al., 2016). In the current work, two immunodominant glycoproteins, BCG_0980 and BCG_0470, were identified using antisera against BCG-immunized mice (as shown in Figure 4). Orthologs of these two glycoproteins (Rv0928 (PstS3) and Rv0431, respectively) were identified in *M. tuberculosis*, and we found that the serum-reactivity of PstS3 was consistent with the previous reports (Baumann et al., 2013, 2015). We have previously shown that mannosylation of Rv0431 may play a role in mediating bacterial immune evasion (Deng et al., 2016). Recently, Rv0431 was reported to be a vesiculogenesis and immune response regulator (virR) involved in regulating the release of *M. tuberculosis*-derived material via the TLR-2 pathway (Rath et al., 2013). However, the current work is the first to identify the potential antigenicity of Rv0431 (BCG_0470).

Several mycobacterial mannoproteins including Apa, LpqH and LprG are involved in host immune regulations. Of these proteins, Apa the best-studied of these proteins, the placement of its mannose modifications have been shown to determine its ability to stimulate a T-lymphocyte response (Horn et al., 1999). Furthermore, Apa mannosyl appendages act as binding sites for host receptors including lung surface protein A and DC-SIGN, and are important for *M. tuberculosis* survival (Pitarque et al., 2005; Ragas et al., 2007). LpqH, another well-studied glycoprotein in *M. tuberculosis*, plays a role in antigen processing. The binding activity of LpqH with host receptors also relies on the integrated mannosyl residues (Herrmann et al., 1996). LprG, a glycoprotein found in *Mycobacterium leprae*, activates MHC II-restricted T-lymphocytes in patients with lepromatous leprosy. Complete mannosylation is required for T-lymphocyte activation (Sieling et al., 2008). Therefore, we hypothesized that the two immunodominant antigens identified in the current work (BCG_0470 and BCG_0980) were potential targets to regulate the immune responses induced by BCG, and that the manosyl appendages of these glycoproteins were essential for high-level antigenicity.

In the present study, mice were immunized with BCG by intravenous injection, which has been shown to elicit stronger immune responses than other routine immunization routes (Darrah et al., 2020). DTH responses *in vivo* along with various immune factors were assessed. We found that the presence of mannose modifications may be responsible for a stronger DTH response. It is well known that a Th1-driven DTH response is associated with protective immunity against mycobacterial infection (Nadler et al., 2003). The detection of DTH-associated cytokines in the footpads of inoculated mice confirmed that the stronger DTH responses were the result of Th1 cytokines such as IFN-γ and TNF-α rather than Th2 cytokine such as IL-4. However, the exact T-regulatory and effector cells involved in the DTH response, as well as the underlying pathway evoked during the response, need to be explored further. Our results also showed that both BCG_0470 and BCG_0980 could induce stronger Th1 responses, including IFN-γ and TNF-α, and stimulate higher concentrations of specific IgG compared with BCG treatment alone. Further investigation is now required to determine whether an immune response is triggered by these two glycoproteins alone.

High level of mannosylation of BCG_0470, which accounted for nearly 7 kDa in molecular mass (Figure 7) was a focus of this study. Because mannosylated protein induced higher levels of BCG_0470-specific IgG in the mice compared with un-mannosylated protein, we concluded that the mannoses oligosaccharides linked with BCG_0470 was an important determinant of B-cell antigenicity. We also analyzed the B-cell Epitopes for BCG_0470 and BCG_0980 using Bepipred Linear Epitope Prediction^2^. As shown in Table 2, there were five common B-cell epitopes (≥4 residues in length) for BCG_0470. In addition, all of the potentially O-mannosylated sites for BCG_0470 were included within one B-cell epitope (Table 2). Thus, it may be possible for the mannose oligosaccharides of BCG-0470 to act as antigenic determinants. Recently, several glycoconjugate vaccines and specific antibodies have been inducibly produced by the carbohydrate-recognizing T cells (Tcars), a subclass of T-helper cells that recognize the carbohydrate of glycopeptides presented by MHC-II molecules, aiding B-cell maturation and specific memory (Sun et al., 2019). Our results regarding BCG_0470-specific IgG production are consistent with the above findings.

**TABLE 2:** Bioinformatic B-cell Epitopic analysis of BCG 0470 and BCG_0980.

**Protein**	**Peptide predicted to be a B-cell epitope (underlined is the potential mannosylated site)**
BCG_0470	8MNERVPDSSGL18 42LGSSPNSEDDSSAISTMTTTTAAPTSTSVKPAAPRA77 85SGAEGAAARTADRL98 125EVEGERATADAVGR138 150ELSDQPPG157
BCG_0980	24GNDDNVTGGGATTGQASAKVDCGGKKTLKASGSTAQAN61 73ACPGQTLNYTANGSGAGISEFNGNQTDFGGSDVPLSKDEAAA AQRRCGSPAW124 161TQWNNP166 189DESGTTDN196 204ASNGAWGKGAGKSFQGGVGEGARGNDGTSAAAKNTPGSIT Y244 262TSAGGDPVAI271 278QTIA281 285ISGV288 299FYRPKRPGSY308 322YPDSQVGT329 339IGAGQSGLGDNGYIPIPDEFKS360

*Common B-cell Epitopes were predicted using Bepipred Linear Epitope Prediction (IEDB Analysis Resource v2.15.1, http://tools.immuneepitope.org). Only the epitope including ≥4 residues in length was considered. The O-mannosylated sites were predicted using NetOglyc predictions (http://www.cbs.dtu.dk/services/NetOGlyc/, NetOGlyc 4.0 Server).*

In summary, we identified a total of 15 O-mannosylated proteins secreted by BCG. Of these, two glycoproteins (BCG_0980 and BCG_0470) were identified as immunodominant glyco-antigens. We found that mannose linkages on both proteins contributed to the stimulation of a T-cell response via DTH *in vivo*, and were necessary for inducing high level of antigenic IgG. These findings imply that mannose linkages maybe the antigenic determinants. Therefore, a further exploration of BCG glycoproteins will contribute to a better understanding of the protective mechanism of the BCG vaccine, allowing the development of subunit vaccine to optimize BCG.

## Data Availability Statement

The datasets generated for this study can be found in the ProteomeXchange (http://www.proteomexchange.org/) (PXD017576).

## Ethics Statement

The animal study was reviewed and approved by the Animal Ethics Committee of the Dalian Medical University.

## Author Contributions

All authors have read and approved the manuscript and contributed significantly to this work. GD, WZ, and SY have contributed to the conception and design, and were in charge of writing. GD, WZ, NJ, and XS performed the experiments. GD, YZ, and XL contributed to the data analysis and figure preparation.

## Conflict of Interest

The authors declare that the research was conducted in the absence of any commercial or financial relationships that could be construed as a potential conflict of interest.

## Footnotes

1 http://www.matrixscience.com

2 http://tools.immuneepitope.org
